# Unusual Localization of Presumptive *Sarcina ventriculi* in the Terminal Ileum: A Case Report

**DOI:** 10.3390/pathogens14090931

**Published:** 2025-09-16

**Authors:** Dua Abuquteish, Daifallah AlNawawi, Reza Khorvash, Osama M. Abu Ata, Nidal Almasri

**Affiliations:** 1 Department of Microbiology, Pathology and Forensic Medicine, Faculty of Medicine, The Hashemite University, Zarqa 13133, Jordan; 2 Department of Pathology and Laboratory Medicine, King Hussein Cancer Center, Amman 11941, Jordan; da.11354@khcc.jo; 3 Faculty of Medicine, The Hashemite University, Zarqa 13133, Jordan; daifallah676@gmail.com; 4 Arizona College of Osteopathic Medicine, Midwestern University, Glendale, AZ 85308, USA; reza.khorvash@midwestern.edu; 5 King Hussein Cancer Center, Amman 11941, Jordan; osamaabuata@yahoo.com

**Keywords:** GVHD, immunocompromised, ileitis, colitis, *Sarcina ventriculi*, Hodgkin Lymphoma

## Abstract

Background: *Sarcina ventriculi* is a bacterium predominantly reported in the stomach and associated with emphysematous gastritis, delayed gastric emptying, gastroparesis, or gastric outlet obstruction. Its prevalence is increasing among patients with a history of organ transplants, immunosuppression, and graft-versus-host disease (GVHD). This bacterium can be detected on histology with characteristic tetrad packet morphology; however, confirmation requires PCR and molecular studies. The role of *Sarcina ventriculi* in human diseases is not fully understood and has unclear clinical significance. While certain studies point to a possible pathogenic role, others regard its detection as incidental with no clear clinical consequence. Case presentation: Herein, we report a case of a 39-year-old male patient with primary refractory cHL, stage IVb, who underwent an autologous bone marrow transplant (BMT) and an allogeneic stem cell infusion. His post-transplant course was complicated by chronic kidney disease (CKD), malnutrition, depression, myopathy, skin, and colon GVHD. He eventually developed sepsis, was admitted to the ICU and developed multiorgan failure and passed away. The patient developed diarrhea, and the gastrointestinal specialist was consulted and revealed ulcerated ileitis and colitis. Biopsies were taken to evaluate for CMV infection and GVHD. The terminal ileum biopsy mainly revealed ulceration with granulation tissue formation and abundant microorganisms arranged in distinctive tetrads, characteristic of *Sarcina ventriculi*. The colonic biopsies were consistent with GVHD grade II. Conclusions: The significance of Sarcina microorganisms and their mechanisms of injury remain poorly understood. The identification of *Sarcina ventriculi* in the terminal ileum, which is an unusual and previously unreported finding, adds a new perspective to our understanding of its pathogenic potential and anatomical distribution. While the patient’s clinical decline was influenced by multiple factors, including GVHD, recurrent sepsis, and multiorgan failure, the role of *Sarcina ventriculi* as a potential exacerbating factor remains unclear.

## 1. Introduction

Classical Hodgkin Lymphoma (cHL) is a hematopoietic malignancy that, despite high cure rates with first-line therapies, poses significant treatment challenges in refractory or relapsed cases [1]. For patients with primary refractory disease, aggressive treatments such as autologous and allogeneic stem cell transplantation are often required [1]. Nevertheless, these interventions carry a high risk of complications, including graft-versus-host disease (GVHD), opportunistic infections, and organ dysfunction. These complications can impact the patient’s prognosis and quality of life [2]. Rare infections such as *Sarcina ventriculi*, a historically underrecognized Gram-positive bacterium, can occur in immunocompromised individuals [3]. *Sarcina ventriculi* has been primarily associated with gastric pathology; however, its identification in the terminal ileum, as in the present case, is extremely uncommon [4,5]. Therefore, we report a patient with primary refractory cHL who underwent an autologous bone marrow transplant (BMT) and was found to have Sarcina microorganisms on a terminal ileum biopsy. This case improves the understanding of rare opportunistic infections in immunocompromised patients with GVHD, such as *Sarcina ventriculi*, offering insights into diagnostic and treatment challenges and the potential clinical implications for these patients.

## 2. Case Report

A 39-year-old male patient with primary refractory classical Hodgkin Lymphoma (cHL), stage IVb, underwent an autologous bone marrow transplant (BMT) and, two years later, received an allogeneic stem cell infusion. His post-transplant course was complicated by chronic kidney disease (CKD), malnutrition, poor oral intake, depression, which was treated with mirtazapine and sertraline, and myopathy that left him nearly bedridden despite physiotherapy. The patient presented to the clinic for routine clinical evaluation post-discharge (discharged 2 days before) and was found to be hypotensive 70/45 mmHg. He was discharged on amlodipine 5 mg bid, carvedilol 12.5 mg bid. Three days later, he presented to the emergency room with generalized fatigue and still had decreased oral intake. On physical examination, he was febrile at 38.5 °C and had tachypnea (respiratory rate 28). Labs showed creatinine 3 mg/dL (was 2 mg/dL on discharge), metabolic acidosis 7.25, and elevated CRP 156 mg/L and procalcitonin 1.68 ng/mL. Thus, he was admitted to the ICU as a case of sepsis and acute, on top of CKD for IV fluids and IV antibiotics.

**Figure 1:**
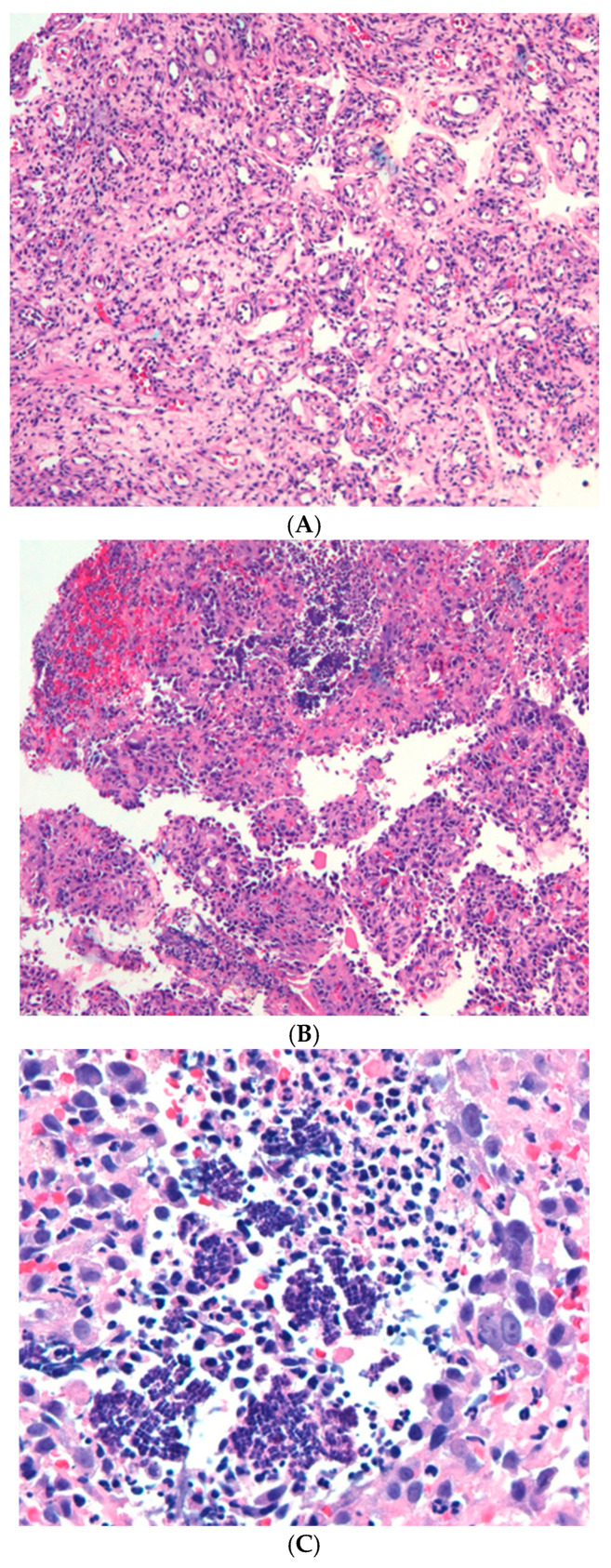
(**A**). H&E staining (10×): terminal ileum biopsy showing ulcer bed and granulation tissue formation. (**B**). H&E staining (10×): terminal ileum biopsy: microorganisms are seen embedded in the granulation tissue. (**C**). H&E staining (40×): high power view showing the tetrads typical for Sarcina microorganism.

During admission, he developed diarrhea 4–5 times daily and a maculopapular rash on areas such as shoulders, palms, and soles—clinically a picture of gut and skin GVHD. The gastrointestinal team was consulted, and upper and lower endoscopy were performed. Stool analysis was performed and found to be negative for microorganisms. Upper endoscopy revealed normal esophageal mucosa and a stomach displaying diffuse gastropathy, while the duodenum appeared normal. A colonoscopy showed ulcerated colitis and ileitis. Biopsies were taken to evaluate for CMV infection and GVHD. The terminal ileum biopsy mainly revealed granulation tissue with abundant microorganisms arranged in distinctive tetrads, characteristic of *Sarcina ventriculi* (Figure 1A–C). The colonic biopsies had increased crypt apoptosis and dropout, consistent with GVHD grade II. Immunostain for CMV and HSV were negative. The patient’s management included high-dose intravenous antibiotics (meropenem, levofloxacin, and vancomycin), along with antifungal and antiviral therapies and interventions for septic shock. While his condition initially stabilized, it later deteriorated, leading to multi-organ failure.

The patient deteriorated with a clinical picture of septic shock, bacteremia, and a chest infection. He failed non-invasive ventilation and was subsequently intubated. He was started on intravenous antibiotics including meropenem, levofloxacin, and vancomycin, as well as foscarnet and micafungin. Additionally, he received albumin, intravenous stress-dose steroids (transitioned from oral), and granulocyte colony-stimulating factor (GCSF).

The patient developed acute kidney injury on top of CKD. He experienced pancytopenia attributed to disease progression, with hemoglobin 6.6, platelets 15, and WBC 0.4. He received two units of PRBCs, one unit of SDP, with follow-up CBC and transfusion as needed. Furthermore, a PCR test confirmed cytomegalovirus (CMV) reactivation, and a liver biopsy showed hemosiderosis. Supportive treatments included mechanical ventilation, renal support, and transfusions for pancytopenia. Unfortunately, his condition worsened, and he eventually developed asystole and passed away.

The three figures below (Figure 1A–C) show histological images of the terminal ileum, highlighting the ulceration, granulation tissue, and the presence of Sarcina microorganisms.

Consent section: Informed consent was obtained from the patient’s parents for publication of this case report and the accompanying images, following the patient’s death.

## 3. Discussion

*Sarcina ventriculi*, a living organism known to exist for more than a century, was discovered by anatomist Goodsir from a patient who had abdominal pain and a unique type of vomiting, which is referred to nowadays as “Sarcinious Vomiting” [6]. Although it is more well-known in veterinary medicine as an infection that affects pets and farm life, there has been a rise in human cases [7,8]. Research suggests that this pathogen was initially found in environmental soil and is transmitted to both animals and humans through the contamination of food and water [7,8]. It is particularly prevalent in the feces of individuals that partake in herbivorous diets [5,7]. Additionally, it has been observed that the infection occurs more frequently in females than in males, at a ratio of 2:1.

The research by Tartaglia, Lam-Himlin, DiMiao, AlRasheed, Propst, Fanaroff, and Tolentino focused on the location of *Sarcina ventriculi*. This bacterium was predominantly found in the gastrointestinal system (88%), particularly in the stomach, and only rarely in other systems: the respiratory system (5%), urinary system (4%), and hematological system (3%) [3,4,5,8,9,10,11]. In this case report, however, the bacteria were discovered in the terminal ileum, despite the pH levels of these organs being 7.4, which typically maintain normal bacterial structures. This finding contrasts with the research conducted by Susan E. Lowe, who reported that at a pH of 7.0, the bacteria appear granulated and undergo apoptosis [12].

*S. ventriculi* was at first known to cause emphysematous gastritis, delayed gastric emptying, and even bleeding in patients who had peptic ulcers, GERD, a history of gastrointestinal surgery, gastroparesis, or gastric outlet obstruction [3]. However, it started to become more profound in patients who have a history of organ transplants, whether it is a kidney or liver, those who are undergoing immunosuppression from immune diseases such as GVHD, or patients who have a history of adenocarcinoma of the stomach [10,13,14].

Sarcina species can be easily recognized in histological studies with their unique tetrad packet. However, it is not specific to Ventriculi, as other bacteria have the same shape, such as *Sarcina maxima*. It is differentiated from its diameter size, where *S. ventriculi* is 2.0 microns, and *S. maxima* is 2.5 microns [15,16]. Another bacterium with a similar morphological structure, but that is completely smaller than them and is catalase negative would be Micrococcus, with a diameter of 0.5 microns [4,17]. Another differentiation is using the Hematoxylin and Eosin (H&E) stain or the silver stain that can mimic a fungal infection [3,7]. Definitive identification requires molecular methods, such as 16S rRNA gene sequencing by PCR [3], which were not available in this case. Importantly, there are no well-documented human infections caused by *S. maxima* in the literature, and nearly all reported cases have been attributed to *S. ventriculi*. Based on the histological findings in our patient, the organism was classified as presumptive *Sarcina ventriculi*. Immunostaining for other microorganisms, such as cytomegalovirus (CMV), yielded negative results.

The gold standard for definitive and precise confirmation of diagnosis involves the use of polymerase chain reaction (PCR) or gene sequencing for the Sarcina 16S ribosomal RNA gene or the pyruvate decarboxylase (PDC) gene [18,19,20]. Unfortunately, these molecular techniques are not available at our center, which prevented us from confirming the diagnosis. As a result, the diagnosis was made solely based on histological morphology. This lack of molecular confirmation is a limitation of our report.

Chen, Liu, and Fu 2025 [21] recently reported two cases of gastric Sacrcina ventriculi infection. One patient presented with small bowel obstruction, while the other experienced epigastric pain. Both cases were diagnosed with histopathology examination. The symptoms in both patients were resolved after treatment, using a combination of antibiotics and a proton pump inhibitor. Also, follow-up examinations showed clearance of *Sarcina ventriculi*. Consequently, they concluded that *Sarcina ventriculi* may have pathogenic potential rather than merely being a benign colonizer.

The role of *Sarcina ventriculi* in human diseases is not fully understood and has an unclear clinical significance, as it has been reported in both symptomatic and asymptomatic patients. While certain studies point to a possible pathogenic role, linking it to severe gastric complications, others regard its detection as incidental with no clear clinical consequence [21,22,23].

Our case represents the first documented instance of presumptive *Sarcina ventriculi* being identified in the terminal ileum. The patient presented with diarrhea, which was explained by the presence of colonic GVHD. However, it remains unclear whether the presence of these microorganisms worsened the patient’s symptoms or if they were merely colonizers, coincidentally found alongside the GVHD. Moreover, it was impossible to determine if the ulceration and granulation tissue in the terminal ileum, which showed no viable epithelium, was solely attributable to the GVHD or if it was caused or exacerbated by the presence of Sarcina. Moreover, an upper endoscopic examination of the patient revealed only diffuse gastropathy, with no distinct signs of inflammation. Stomach biopsies showed chronic inactive gastritis, no increase in epithelial apoptosis, and no identified microorganisms.

Pharmacological therapy for *S. ventriculi* would include the usage of metronidazole with an antibacterial such as ciprofloxacin with the option to add a Proton Pump Inhibitor (PPI) [3]. Despite that being a treatment, there is no agreement on the regimen and the time required to fully eradicate the microorganism since there is not enough data for any follow-up to see its status [5]. AlRasheed’s study also showed that out of the 19 cases that had the bacterium, 7 received antimicrobial therapy, 2 received PPI only, 2 underwent surgery, and the remainder did not have to take any medications for the disease, giving the topic room to be debated further [3]. Our patient, in this case, had a complicated course with bacteremia and sepsis and received multiple antibiotics regimens. The presence of Sarcina in this complicated case with colonic GVHD made it unclear whether the bacteria caused the diarrhea and the terminal ileum ulceration with granulation tissue, or if it was due to severe GVHD.

## 4. Conclusions

The significance of Sarcina microorganisms and their mechanisms of injury have not been fully understood. The identification of presumptive *Sarcina ventriculi* in the terminal ileum, which is an unusual location for this microorganism, adds a new perspective to our understanding of its pathogenic potential and anatomical distribution. Although the patient’s clinical deterioration was driven by several factors, including GVHD, recurrent sepsis, and reactivation of CMV, the role of *Sarcina ventriculi* as a potential exacerbating factor remains uncertain. This report emphasizes the importance of histopathological examination and a multidisciplinary approach in managing critically ill patients. Further research is necessary to clarify and understand the pathogenicity of *Sarcina ventriculi* in immunocompromised individuals and to establish standardized treatment protocols.

## Data Availability

Data are contained within the article.
